# Narrow band imaging under less-air condition improves the visibility of superficial esophageal squamous cell carcinoma

**DOI:** 10.1186/s12876-020-01534-2

**Published:** 2020-11-19

**Authors:** Taro Iwatsubo, Ryu Ishihara, Yasushi Yamasaki, Yusuke Tonai, Kenta Hamada, Minoru Kato, Sho Suzuki, Mitsuhiro Kono, Hiromu Fukuda, Yusaku Shimamoto, Kentaro Nakagawa, Masayasu Ohmori, Masamichi Arao, Kenshi Matsuno, Hiroyoshi Iwagami, Shuntaro Inoue, Hiroko Nakahira, Noriko Matsuura, Satoki Shichijo, Akira Maekawa, Takashi Kanesaka, Yoji Takeuchi, Koji Higashino, Noriya Uedo, Kazuhide Higuchi

**Affiliations:** 1 Department of Gastrointestinal Oncology, Osaka International Cancer Institute, 3-1-69 Otemae, Chuo-ku, Osaka, 541-8567 Japan; 2 Second Department of Internal Medicine, Osaka Medical College, Osaka, Japan; 3 Department of Gastroenterology, Okayama University Hospital, Okayama, Japan; 4 Department of Gastroenterology, Sanda City Hospital, Hyogo, Japan; 5 Department of Gastroenterology and Hepatology, Osaka University Graduate School of Medicine, Osaka, Japan; 6 Division of Endoscopy and Center for Digestive Disease, Department of Gastroenterology and Hepatology, University of Miyazaki Hospital, Miyazaki, Japan; 7 Department of Gastroenterology, Takayama Red Cross Hospital, Gifu, Japan

**Keywords:** Narrow band imaging, Less air, Superficial esophageal squamous cell carcinoma

## Abstract

**Background:**

The current virtual chromoendoscopy equipment cannot completely detect superficial squamous cell carcinoma (SCC) in the esophagus, despite its development in the recent years. Thus, in this study, we aimed to elucidate the appropriate air volume during endoscopic observation to improve the visibility of esophageal SCC.

**Methods:**

This retrospective study included a total of 101 flat type esophageal SCCs identified between April 2017 and January 2019 at the Department of Gastrointestinal Oncology, Osaka International Cancer Institute. Video images of narrow band imaging (NBI) under both less-air and standard-air conditions were recorded digitally. Videos were evaluated by five endoscopists. Relative visibility between less-air and standard-air conditions of the brownish area, brownish color change of the epithelium, and dilated intrapapillary capillary loop (IPCL) were graded as 5 (definitely better under less-air condition) to 1 (definitely worse under less-air condition), with 3 indicating average visibility (equivalent to standard-air observation).

**Results:**

The mean (standard deviation) visibility score of the brownish area, brownish color change of the epithelium, and dilated IPCLs under less-air condition were 3.94 (0.58), 3.73 (0.57), and 4.13 (0.60), respectively, which were significantly better than that under standard-air condition (*p* < 0.0001). Esophageal SCC evaluated as ≥ 4 in the mean visibility score of the brownish area, brownish color change of the epithelium, and dilated IPCLs accounted for 50% (51/101 lesions), 34% (34/101 lesions), and 67% (68/101 lesions), respectively.

**Conclusions:**

The present results suggested that NBI with less air might improve the visibility of flat type esophageal SCC compared with NBI with standard air. Less-air NBI observation may facilitate the detection of flat type esophageal SCC.

***Trial registration*:**

The present study is a non-intervention trial.

## Background

Esophageal cancer is the sixth most common cause of cancer-related mortality worldwide [1]. In Japan, more than 20,000 new cases develop [2] and more than 10,000 patients die annually [3], of which squamous cell carcinoma (SCC) accounts for 88% of the cases. Although advanced-stage esophageal cancers are rarely curable, early-stage cancers have a good prognosis; the 5-year survival rate of patients treated with endoscopic resection was 86.0% [4]. Therefore, early detection of esophageal cancer is essential to improve the prognosis.

For early detection of esophageal SCC, iodine solution had been used as the most sensitive modality [5]. This method, however, causes severe mucosal irritation leading to retrosternal pain and discomfort. Therefore, this method is less recommended for screening endoscopy. In recent years, endoscopic technology has been dramatically developing. Virtual chromoendoscopy, including narrow-band imaging (NBI) and blue laser imaging (BLI), has enabled early detection of esophageal superficial SCC [6–8]. In experienced hands, the sensitivity of NBI to detect esophageal SCC was comparable with that of iodine solution which has been accepted as the gold standard modality for detection of esophageal SCC [8–10]. Furthermore, NBI observation with magnification enables prediction of cancer invasion depth by evaluating the shape of the intrapapillary capillary loop (IPCL) of an esophageal neoplasm [11].

Despite its high sensitivity, some concerns remain regarding the use of NBI for the detection of esophageal SCC. For example, the sensitivity of NBI to detect esophageal SCC among inexperienced endoscopists was lower than that of chromoendoscopy with iodine solution [12]. Some SCCs could not be identified even by experienced endoscopist until iodine spraying [8, 13]. To overcome such shortcomings, standardization of the observing condition by NBI is required. Tension of the esophageal wall is one of the most important factors that determine the quality of esophageal observation, since the visibility of esophageal SCC changes by inflation or suction of air. However, the preferred condition regarding air is not determined yet. Thus, we aimed to investigate whether controlling the air during observation (standard air or less air) influences visibility of the esophageal SCC.

## Methods

### Study design and population

This was a single-center retrospective study conducted between April 2017 and January 2019 at the department of gastrointestinal oncology of a cancer institute. The study protocol was approved by the institutional review board of the cancer institute (no.18222).

Patients recently diagnosed with esophageal SCC scheduled for work-up, endoscopic treatment, or surveillance endoscopy after endoscopic resection for esophageal SCC were selected as candidate study subjects. Patients with a history of radiotherapy for esophageal cancer and those with multiple lesions were excluded from candidate study subjects.

Endoscopic observation of esophageal SCC by NBI was conducted with sufficient air (standard air) condition, which is the usual condition for observing the esophagus, and less-air condition (Additional file 1: Video). We started this procedure since April 2017 and recorded digitally all procedures. For less-air condition, we suctioned the air in the esophagus to maintain a minimal lumen during observation of the esophageal mucosa, and mild magnifying function was used to adjust the focus. All procedures were performed by endoscopists who were familiar with this procedure or who pre-learned this procedure using the reference video prior to recording the procedure. The following endoscopies were used: RQ260Z, GIF-Q240Z, H260Z, H290Z, and H290EC with the EVIS LUCERA ELITE endoscopic system (Olympus Medical Systems Co., Tokyo, Japan). Although high magnification was never required under less-air condition, a soft black hood (MB162 or MB46; Olympus Co.) was routinely attached to the tip of the endoscope in case high magnification observation is necessary. After the observation, biopsy or endoscopic resection was performed as necessary.

The recorded video during the study period that met the following criteria were retrieved: histologically confirmed esophageal SCC, lesion size < 20 mm by endoscopic observation, and 0-II type morphology by the Paris classification. We excluded 0-I and 0-III types because they are easily identified by endoscopic observation [14].

### Histological evaluation

Biopsy or endoscopically resected specimens were embedded in paraffin and subjected to hematoxylin and eosin staining. Histological diagnosis and depth of tumor invasion was diagnosed according to Japanese Classification of Esophageal Cancer, 11th Edition [15, 16]. According to the criteria, “carcinoma” was defined on the basis of the architectural and cytological changes observed without morphological evidence of invasive growth. Therefore, SCC includes tumor confined to the epithelium, which is an indication for endoscopic resection in Japan [17].

### Evaluation of endoscopic images

**Fig. 1:**
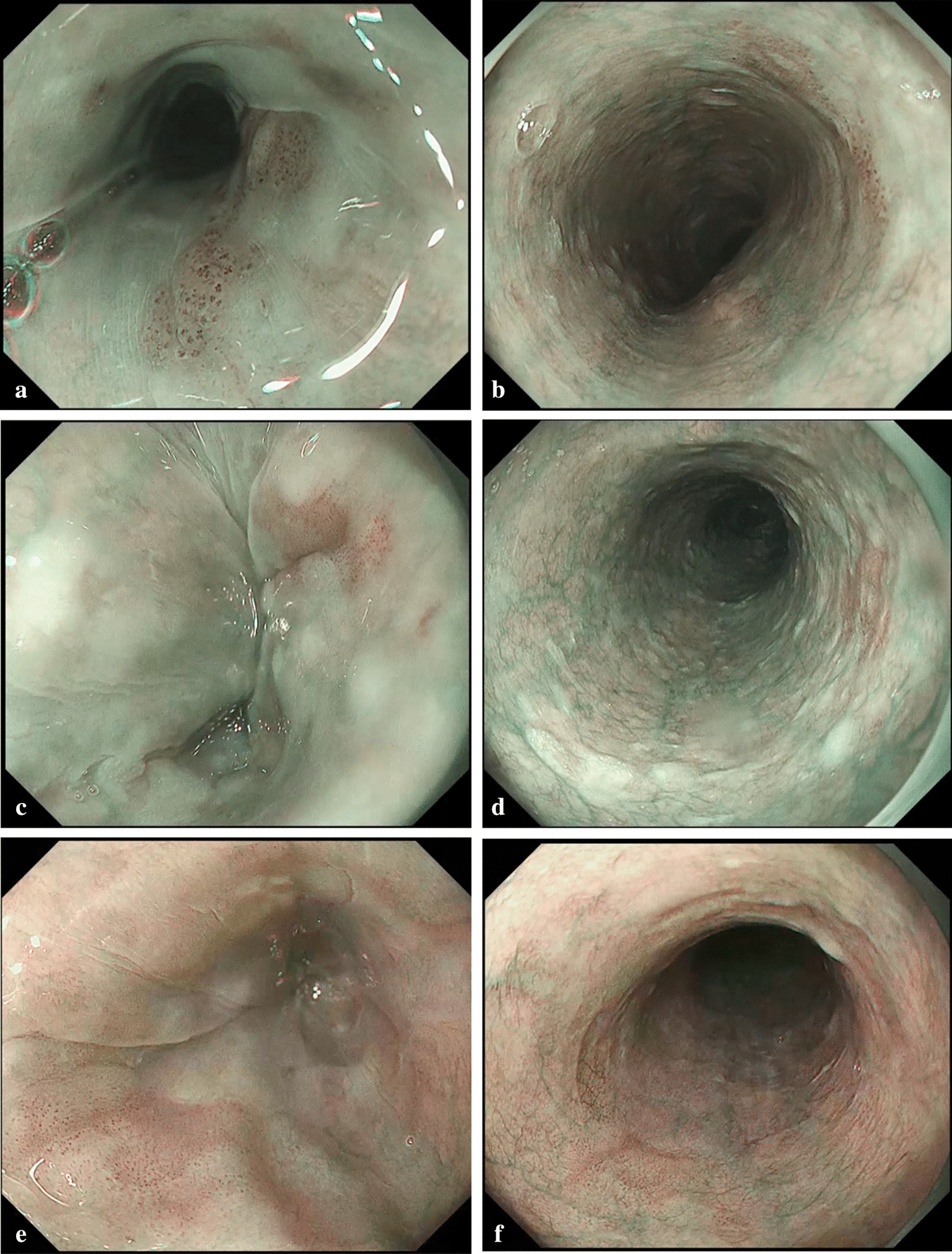
Visibility scores of superficial esophageal squamous cell carcinoma with NBI under less-air and standard-air conditions. **a** Narrow band imaging (NBI) under less-air condition: visibility score, 5. **b** NBI under standard-air condition: visibility score, 5. **c** NBI under less-air condition: visibility score, 4. **d** NBI under standard-air condition: visibility score, 4. **e** NBI under less-air condition: visibility score, 3. **f** NBI under standard-air condition: visibility score, 3. *NBI* narrow band imaging

The primary outcome was visibility of the identified SCC and a well-demarcated brownish area, which is the most representative finding of SCC by NBI. Visibility of SCC was evaluated according to the following interval scale (Fig. 1): 5 (excellent visibility), definitely better visibility by less-air than by standard-air observation; 4 (good visibility), better visibility by less-air than by standard-air observation (intermediate scale between scores 3 and 5); 3 (average visibility), the same visibility by less-air as standard-air observation; 2 (fair visibility), worse visibility by less-air than by standard-air observation (intermediate scale between scores 1 and 3); and 1 (poor visibility), definitely worse visibility by less-air than standard-air observation. Besides, we evaluated the visibility of (1) a brownish color change of the epithelium [18, 19] and (2) dilated IPCLs [20] according to the above visibility scores as well; the brownish area of esophageal cancer constitutes a brownish color change of the epithelium and dilated IPCLs. Evaluation was performed by five endoscopists who never know the study purpose and had not viewed any of these videos before this study. All five experienced endoscopists were board-certified specialists at the Japan Gastroenterological Endoscopy Society. Each video including standard-air or less-air condition were created by endoscopist "TI" other than the five evaluators so that the length would become around 10 s. The order and the number of times of reviewing videos were not limited because of short video.

### Statistical analysis

Continuous variables are presented as the mean and standard deviation (SD) or median and range, as appropriate for the data type. Five phases of the evaluation (lesion visibility scores 1–5) were used as an interval scale; for comparisons between methods (less air and standard air), Wilcoxon signed-rank test was used. Univariate analysis of the association between excellent or good visibility of the brownish area (mean visibility score ≥ 4.0) and each endoscopic finding was performed using chi-squared test and Fisher’s exact test for comparisons of variables. Probability values for statistical tests were two-tailed and *p* < 0.05 was considered significant. All analyses were performed using the statistical program “R” version 3.3.3 (R Foundation, Vienna, Austria).

## Results

A total of 103 lesions were candidates for the study. Among them, video images of two lesions were of low quality because of mucus or severe peristalsis; hence, they were excluded from the assessment. Finally, a total of 101 lesions in 101 patients were included in the analysis. Closer view and mild magnification were applied for all of the lesions under less-air condition. Moreover, even by magnified observation, there were neither extremely bright or dark view and tangential view.

The characteristics of the patients and lesions are summarized in Table 1. Of 101 lesions, five were morphologic type 0-IIa, 37 were type 0-IIb, and 59 were type 0-IIc. The median lesion size was 12 mm (range 3–20 mm). Four lesions were histologically diagnosed by biopsy specimens, and 97 lesions were diagnosed by endoscopically resected specimens (35 lesions by EMR and 62 lesions by ESD).

**Table 1:** Characteristics of the enrolled patients

	Total (n = 101)
Sex (male/female), n	82/19
Age, median (range), years	69 (43–84)
Location, n	
Anterior/posterior/right/left	18/45/17/21
Ce/Ut/Mt/Lt/Ae	2/15/67/17/0
Morphology, n (%)	
0-IIa	5 (5)
0-IIb	37 (37)
0-IIc	59 (58)
Endoscopic lesion size, median (range), mm	12 (3–20)
Final histological diagnosis, n (%)	
Biopsy	4 (4)
EMR/ESD	35 (35)/62(61)
Depth of invasion, n (%)	
pT1a-EP	25 (26)
pT1a-LPM	69 (68)
pT1a-MM	2 (2)
pT1b-SM1	0 (0)
pT1b-SM2	1 (1)
Unknown	4 (4)

*Ce* cervical esophagus, *EMR* endoscopic mucosal resection, *EP* epithelium, *ESD* endoscopic submucosal dissection, *LPM* lamina propria mucosae, *Lt* lower thoracic esophagus, *MM* muscularis mucosae, *Mt* middle thoracic esophagus, *SM* submucosa, *Ut* upper thoracic esophagus

**Fig. 2:**
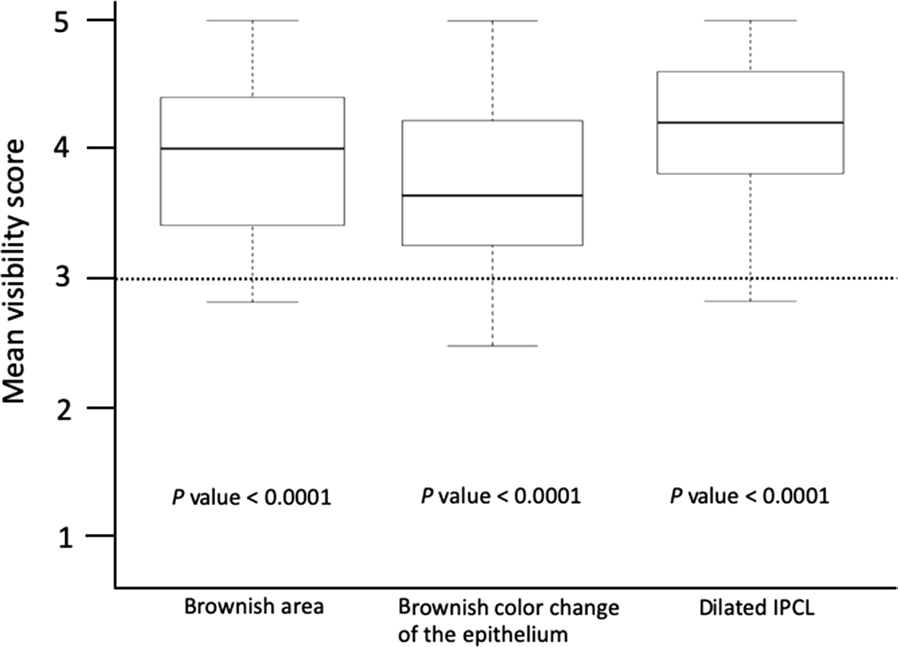
Visibility score under less-air observation compared with under standard-air condition (score 3). *IPCL* intra-epithelial capillary loop

**Fig. 3:**
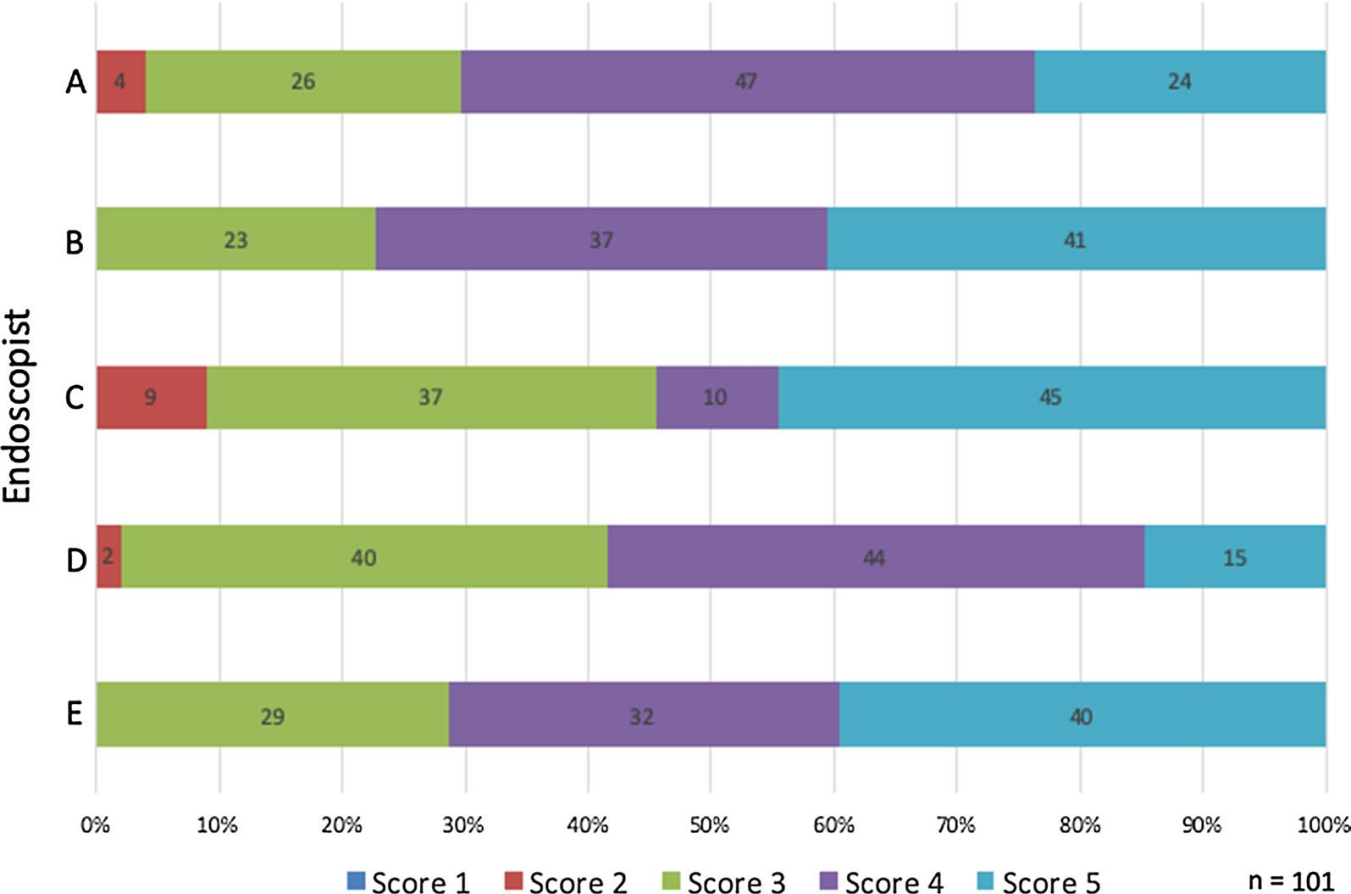
The ratio of visibility score under less-air observation for the brownish area among endoscopists

Visibility scores of NBI under less-air condition are shown in Fig. 2. The mean visibility score of the brownish area under less-air condition was 3.94 ± 0.58 which was significantly better than that under standard-air condition (*p* < 0.0001). The mean visibility scores of the brownish color change of the epithelium and dilated IPCLs under less-air condition were 3.73 ± 0.57 and 4.13 ± 0.60, respectively, which were significantly better than that under standard-air condition (*p* < 0.0001). Esophageal SCC with mean visibility score ≥ 4 considering the brownish area, brownish color change of the epithelium, and dilated IPCLs accounted for 50% (51/101 lesions), 34% (34/101 lesions), and 67% (68/101 lesions) of the lesions, respectively. The rate of visibility scores for the brownish area among five endoscopists are shown in Fig. 3. No lesion was regarded as having poor visibility (visibility score 1) by any endoscopists, and lesions in average, good, and excellent visibility (mean visibility score ≥ 3) accounted for 98% (99/101 lesions) of all esophageal SCC.

The results of the univariate analysis are shown in Table 2. Excellent/good visibility score in the brownish color change of the epithelium and dilated IPCLs was the factor that was significantly associated with excellent/good visibility score in the brownish area (*p* < 0.0001).

**Table 2:** Univariate analysis of predictor for visibility of the brownish area under less-air condition

	Brownish area		*p* value
	Excellent/good ≥ Score 4.0, n = 51	Average/fair/poor < Score 4.0, n = 50	
Visibility score of brownish color change of the epithelium, n			
Excellent/good ≥ score 4.0	34	0	< 0.0001
Average/fair/poor < score 4.0	17	50	
Visibility score of dilated IPCLs, n			
Excellent/good ≥ score 4.0	46	21	< 0.0001
Average/fair/poor < score 4.0	5	29	
Macroscopic type, n			
0-IIa	1	4	0.207
0-IIb	22	15	
0-IIc	28	31	
Lesion size, n			
> 1.0 cm	26	25	1.000
≤ 1.0 cm	25	25	
Location, n			
Ce	2	0	0.219
Ut	9	8	
Mt	30	37	
Lt	10	5	
Cross-sectional circumference, n			
Anterior wall	9	9	0.428
Posterior wall	19	26	
Right wall	10	7	
Left wall	13	8	
Depth, n			
EP	12	13	1.000
LPM	34	35	
MM	1	1	
SM	1	0	

*Ce* cervical esophagus, *EP* epithelium, *IPCL* intrapapillary capillary loop, *LPM* lamina propria mucosae, *Lt* lower thoracic esophagus, *MM* muscularis mucosae, *Mt* middle thoracic esophagus, *SM* submucosa, *Ut* upper thoracic esophagus

## Discussion

Of esophageal SCCs, 50% (51/101 lesions) have mean visibility score ≥ 4.0, 48% esophageal SCCs (48/101 lesions) scored 3.0 to less than 4.0, while the other two (2%) scored 2.8. The present results suggest that less-air condition improved the mean visibility score in more than half of the SCCs and did not worsen in almost all of them.

For the detection of esophageal SCC, chromoendoscopy with iodine solution had been regarded as the standard modality given its high sensitivity [5, 9]. However, this method has several disadvantages such as low specificity, iodine allergy, retrosternal pain, and discomfort caused by mucosal irritation. Therefore, this method is less recommended for screening endoscopy in patient with low risk of esophageal SCC.

NBI enhances the surface structure and microvascular patterns and offers a valid, alternative tool for early detection of esophageal SCC because of its high performance [7, 8]. However, NBI has some limitations; first, the visibility of SCCs is influenced by the observing condition. The light intensity in NBI is low and can be easily scattered in vivo. Especially, detecting lesions from afar is quite challenging using NBI given its limited light intensity. The brightness of NBI was recently improved by the new-generation video processor system (EVIS LUCERA ELITE; Olympus Medical Systems Co.) in comparison with the previous system (EVIS LUCERA SPECTRUM; Olympus Medical Systems Co.) [21]. The new-generation video processor system provides the second-generation (brighter) NBI. All cases in our study equally benefited from the second-generation NBI. Nevertheless, the field of view of NBI is still darker than that of conventional imaging. Second, the endoscopists are required to have enough skill to achieve good observing condition to detect SCC. Thus, the reported sensitivity of NBI for the detection of esophageal SCC is lower among inexperienced endoscopists [12]. To overcome such limitation, a novel method of observing the esophagus for SCC detection is required.

In the selection of the observation condition, controlling the amount of air is a fundamental but important point. It can change the brightness of the area, distance, and angle of the lesions from the endoscope. This study demonstrated that esophageal observation with less air had significantly higher visibility scores than observation with standard air. However, the mechanism of this improved visibility is not yet elucidated. Accordingly, we made some hypotheses for this phenomenon. The most representative finding of esophageal SCC is the brownish area by NBI. The brownish area usually consists of the brownish color change and dilated IPCLs of the cancerous area. This brownish color change of the epithelium is mainly caused by the decreased thickness of the surface keratinous layer [19]. Squamous epithelium has high reflectance [22] and therefore appears brighter than the columnar epithelium in NBI. This property may be derived from the keratinous layer [18, 22]. Suctioning the air from the esophageal lumen thickened the esophageal wall. With thickening of the esophageal wall, the normal esophagus appears brighter (more whitish) because of the thickening of the keratinous layer, while the brownish color change in the cancerous area remains unchanged since the keratinous layer is usually absent in the cancerous area. Therefore, the invasion depth of the lesion might not significantly influence on the difference of its own visibility between each air condition. The distribution of dilated IPCLs, which is another important factor of brownish area, may be also influenced by the amount of air in the esophagus. Dilated IPCLs usually exist in the cancerous area. The distribution of dilated IPCLs becomes denser as the esophageal wall shrunk. Denser distribution of dilated IPCLs in the cancerous area (more brownish) and thickened keratinous layer in the surrounding mucosa (more whitish) under less-air condition may be the main factors contributing to improved visibility of cancer. Second, NBI produces dark image owing to its weak light intensity. This limitation becomes obvious in large observation area, such as the stomach. Suctioning the air and shrinking the esophageal lumen may limit the space and enhance the brightness of the visual field. Increased brightness of the area may enhance the visibility of esophageal lesions. In this study, the second-generation (brighter) NBI was applied for all cases. These findings indicated even the brightness of second-generation NBI might be improved under less-air condition. Third, close observation of the esophageal mucosa by shrinking the esophagus may produce a clearer image of the cancer. Dilated IPCLs and brownish color change are usually very faint findings and sometimes recognizable only by close-up view.

This study has some limitations. First, this is a retrospective analysis of video images taken during endoscopic examination for esophageal SCC. The assessment of detection was unable from study methods because our videos were created for around only lesions. Therefore, the assessment of the visibility was focused on in our study. Usually, real-time analyses of endoscopic procedure provide more realistic information on the utility of endoscopy. However, the assessment of visibility may be largely biased if the evaluators know the clinical question of the study (Does less-air observation enhance the visibility of esophageal SCC?). We, therefore, recorded video images that were assessed by evaluators who did not know the clinical question. By this method, we could avoid such bias of evaluators. Second, the study subjects were limited to patients with flat esophageal SCC (0-II type in the Paris classification). Limiting the morphology of esophageal SCC may impair generalizability of this method. However, esophageal cancers other than 0-II type (i.e., advanced cancer), 0-I, or 0-III type, are easy to detect. Special technique is not required for the detection of esophageal SCC other than 0-II type. Third, video recording of less-air observation was conducted by experienced endoscopists. Although the feasibility of this observation by less experienced endoscopists should be evaluated in the future, the technique can be mastered easily. Fourth, mild magnification was used to adjust the focus for observation under less-air condition. Using mild magnification under the standard-air condition is not feasible because when the scope is at the center of the esophageal lumen, the mucosal surface would be out of focus. Furthermore, attempting to adjust the distance for mild magnification would necessitate close observation of the circumferential mucosa, which would extend the procedure time. However, the latest endoscope (GIF-EZ1500, Olympus Co.) and endoscopic system (EVIS X1, Olympus Co.) have a wide range of focal distances under non-magnified observation, with the closest distance being 3 mm. Thus, the adjustment of focus applied in this study will no longer be required in the future. Fifth, this observation process may extend the examination by 1 or 2 min. However, patients’ discomfort may not be much because less-air observation is achieved without introducing much air and without extending the esophagus. Sixth, in the present study, NBI was used for all cases as virtual chromoendoscopy. A previous study reported that the BLI was comparable with NBI for recognition of esophageal SCC [6]. Therefore, BLI as well as NBI might improve the visibility of esophageal SCC under less-air condition.

## Conclusions

The present study has demonstrated that the NBI with less air improved or maintained the visibility of brownish area compared with the NBI with standard air in flat type esophageal SCCs. This less-air NBI technique may facilitate the detection of SCC in the esophagus, and further studies are necessary in the future for the assessment of the detection.

## Supplementary information

**Additional file 1: Video**. Recorded video image using narrow band imaging (NBI) under less-air following standard-air condition. A superficial esophageal squamous cell carcinoma, 13 mm in size, is located at the right wall of the upper thoracic esophagus.

## Data Availability

The datasets used and/or analysed during the current study are available from the corresponding author on reasonable request.

## Abbreviations

SCC: Squamous cell carcinoma
NBI: Narrow band imaging
BLI: Blue laser imaging
IPCL: Intrapapillary capillary loop

## References

[1] Ferlay J, Soerjomataram I, Dikshit R, Eser S, Mathers C, Rebelo M (2015). Cancer incidence and mortality worldwide: sources, methods and major patterns in GLOBOCAN 2012. Int J Cancer.

[2] Hori M, Matsuda T, Shibata A, Katanoda K, Sobue T, Nishimoto H (2015). Cancer incidence and incidence rates in Japan in 2009: a study of 32 population-based cancer registries for the Monitoring of Cancer Incidence in Japan (MCIJ) project. Jpn J Clin Oncol.

[3] Vital Statistics Japan (Ministry of Health, Labour and Welfare). Cited 27 August 2019. https://ganjoho.jp/reg_stat/statistics/index.html.

[4] Tachimori Y, Ozawa S, Numasaki H, Ishihara R, Matsubara H, Muro K (2018). Comprehensive registry of esophageal cancer in Japan, 2011. Esophagus.

[5] Hashimoto CL, Iriya K, Baba ER, Navarro-Rodriguez T, Zerbini MC, Eisig JN (2005). Lugol's dye spray chromoendoscopy establishes early diagnosis of esophageal cancer in patients with primary head and neck cancer. Am J Gastroenterol.

[6] Tomie A, Dohi O, Yagi N, Kitae H, Majima A, Horii Y (2016). Blue laser imaging-bright improves endoscopic recognition of superficial esophageal squamous cell carcinoma. Gastroenterol Res Pract.

[7] Muto M, Minashi K, Yano T, Saito Y, Oda I, Nonaka S (2010). Early detection of superficial squamous cell carcinoma in the head and neck region and esophagus by narrow band imaging: a multicenter randomized controlled trial. J Clin Oncol.

[8] Nagami Y, Tominaga K, Machida H, Nakatani M, Kameda N, Sugimori S (2014). Usefulness of non-magnifying narrow-band imaging in screening of early esophageal squamous cell carcinoma: a prospective comparative study using propensity score matching. Am J Gastroenterol.

[9] Morita FH, Bernardo WM, Ide E, Rocha RS, Aquino JC, Minata MK (2017). Narrow band imaging versus lugol chromoendoscopy to diagnose squamous cell carcinoma of the esophagus: a systematic review and meta-analysis. BMC Cancer.

[10] Goda K, Dobashi A, Yoshimura N, Kato M, Aihara H, Sumiyama K (2015). Narrow-band imaging magnifying endoscopy versus Lugol chromoendoscopy with pink-color sign assessment in the diagnosis of superficial esophageal squamous neoplasms: a randomised noninferiority trial. Gastroenterol Res Pract.

[11] Oyama T, Inoue H, Arima M, Momma K, Omori T, Ishihara R (2017). Prediction of the invasion depth of superficial squamous cell carcinoma based on microvessel morphology: magnifying endoscopic classification of the Japan Esophageal Society. Esophagus.

[12] Ishihara R, Takeuchi Y, Chatani R, Kidu T, Inoue T, Hanaoka N (2010). Prospective evaluation of narrow-band imaging endoscopy for screening of esophageal squamous mucosal high-grade neoplasia in experienced and less experienced endoscopists. Dis Esophagus.

[13] Dobashi A, Goda K, Furuhashi H, Matsui H, Hara Y, Kamba S (2019). Diagnostic efficacy of dual-focus endoscopy with narrow-band imaging using simplified dyad criteria for superficial esophageal squamous cell carcinoma. J Gastroenterol.

[14] Participants in the Paris Workshop (2003). The Paris endoscopic classification of superficial neoplastic lesions: esophagus, stomach, and colon: November 30 to December 1, 2002. Gastrointest Endosc.

[15] Japan Esophageal Society (2017). Japanese Classification of Esophageal Cancer, 11th Edition: part I. Esophagus.

[16] Japan Esophageal Society (2017). Japanese Classification of Esophageal Cancer, 11th Edition: part II and III. Esophagus.

[17] Kitagawa Y, Uno T, Oyama T, Kato K, Kato H, Kawakubo H (2019). Esophageal cancer practice guidelines 2017 edited by the Japan Esophageal Society: part 1. Esophagus.

[18] Minami H, Isomoto H, Inoue H, Akazawa Y, Yamaguchi N, Ohnita K (2014). Significance of background coloration in endoscopic detection of early esophageal squamous cell carcinoma. Digestion.

[19] Kanzaki H, Ishihara R, Ishiguro S, Nagai K, Matsui F, Yamashina T (2013). Histological features responsible for brownish epithelium in squamous neoplasia of the esophagus by narrow band imaging. J Gastroenterol Hepatol.

[20] Muto M, Nakane M, Katada C, Sano Y, Ohtsu A, Esumi H (2004). Squamous cell carcinoma in situ at oropharyngeal and hypopharyngeal mucosal sites. Cancer.

[21] Ogiso K, Yoshida N, Siah KT, Kitae H, Murakami T, Hirose R (2016). New-generation narrow band imaging improves visibility of polyps: a colonoscopy video evaluation study. J Gastroenterol.

[22] Wilmink GJ, Ibey BL, Tongue T, Schulkin B, Laman N, Peralta XG (2011). Development of a compact terahertz time-domain spectrometer for the measurement of the optical properties of biological tissues. J Biomed Opt.

